# Comparison of 5% Phenol With Almond Oil Versus 15% Hypertonic Saline in Treatment of Pediatric Idiopathic Rectal Prolapse

**DOI:** 10.7759/cureus.23552

**Published:** 2022-03-27

**Authors:** Ghulam Mustafa, Ali Asad, Sidra tul Muntaha

**Affiliations:** 1 Pediatric Surgery, University of Child Health Sciences, The Children's Hospital, Lahore, PAK; 2 Pediatric Surgery, Services Hospital Lahore, Lahore, PAK

**Keywords:** recurrence, 5% phenol in almond oil, 15% hypertonic saline, rectal prolapse, idiopathic

## Abstract

Objective: The objective of the study was to compare the frequency of recurrence with 5% phenol in almond oil versus 15% hypertonic saline for pediatric idiopathic rectal prolapse.

Methodology: An open-label, randomized clinical trial was conducted at the Department of Paediatric Surgery, Services Hospital, Lahore, Pakistan, over a period of one year from May 1, 2018 to April 30, 2019. Altogether, 120 patients with idiopathic rectal prolapse were included in this study. After obtaining approval from the hospital ethical committee, all patients fulfilling the inclusion criteria were admitted to the pediatric surgery inpatient department of Services Hospital, Lahore. Patients were randomized into two groups with an equal number of candidates using the lottery method. Group A consisted of patients who were administered 5% phenol in almond oil and group B consisted of patients who were administered 15% hypertonic saline. All procedures were performed by a single surgical operating team to control bias. Patients were followed up for three months after surgery to note whether recurrence occurred or not.

Results: The mean age of the patients was 3.97 ± 2.68 years in group A and 2.87 ± 1.84 years in group B. Gender distribution showed male dominance (71.7% in group A and 73.3% in group B). Statistically significant difference was observed in terms of recurrence (50% in group A and 23.3% in group B) (p=0.002), while statistically insignificant differences were found in terms of postoperative faecal incontinence (2% in each group, p=0.6478) and anal stenosis (8% in group A and 2% in group B with p=0.2426).

Conclusion: Thus, 15% hypertonic saline was noted to be a more effective sclerosing agent than 5% phenol in almond oil in the management of idiopathic rectal prolapse in children. It was also found to have a statistically comparable rate of complications, including fecal incontinence and anal stenosis.

## Introduction

Rectal prolapse is defined as extrusion of a part of or the entire rectum from the anal canal. The peak incidence of rectal prolapse in children is between the ages of one and three years [1]. Rectal prolapse may be partial, which involves only the mucosa, or complete, which involves extrusion of all the layers [1,2]. According to its etiology, rectal prolapse can be classified into idiopathic and secondary types. In idiopathic rectal prolapse, the cause of prolapse is unknown. However, secondary rectal prolapse is caused by a known pathology, e.g., meningomyelocele. Idiopathic rectal prolapse is usually partial, whereas secondary rectal prolapse has a full thickness.

Rectal prolapse in children results in psychological trauma to the parents as well as the children [3]. Various treatment modalities have been described. Approximately 50% cases of idiopathic rectal prolapse resolve with conservative management, including manual reduction, proper toilet training, and dietary modification [4]. However, sclerotherapy or surgical interventions may be required in cases that fail to respond to conservative management. Surgical intervention could range from perineal procedures to abdominal rectopexies. Owing to high morbidity, these complicated procedures are less favorable for infants and children [5]. Injection sclerotherapy is the recommended treatment alternative in infants and children with idiopathic rectal prolapse. Some commonly used sclerosing agents include sodium tetradecyl sulfate, phenol (5%) in almond oil, ethanolamine oleate, 50% dextrose, hypertonic saline (15% and 30%), cow’s milk, and ethyl alcohol. Injection sclerotherapy is a simple, cost-effective procedure with less complications. In fact, it is a day-care procedure [6,7].

Hypertonic saline has a low complication rate, is easy to inject, and is cost effective. Because it is a saline solution, no hazardous effects are likely to occur if some amount is absorbed in the body [8]. A study conducted by Abes and Sarihan showed no recurrence in patients treated using 15% hypertonic saline [9]. In a local study conducted by Chaudhry et al., 5% phenol and 15% hypertonic saline were compared and the rate of recurrence was found to be 24% in the phenol group and 46% in the 15% hypertonic saline group [10]. Although several studies have been conducted to assess the efficacy of 5% phenol in almond oil, only one study has assessed the efficacy of 15% hypertonic saline solely in rectal prolapse that showed no recurrence. Another study comparing 5% phenol in almond oil versus 15% hypertonic saline in children showed 46% recurrence in the 15% hypertonic saline group, which was relatively high. Because only one study has compared the efficacy of 5% phenol in almond oil versus 15% hypertonic saline in pediatric cases of rectal prolapse in terms of recurrence rate, more studies are required to perform adequate comparison. This study addresses this concern.

## Materials and methods

Study design

An open-label, randomized controlled trial was performed at the Department of Paediatric Surgery, Services Hospital, Lahore, Pakistan, over a period of six months. A sample size of 120 (60 in each group) participants was calculated at a 5% level of significance and 80% power of test. The expected frequency was 24% for 5% phenol and 46% for 15% hypertonic saline.

Inclusion and exclusion criteria

Children of either gender in the age group of one to 12 years with a diagnosis of idiopathic rectal prolapse were included in this study. Failed cases after sclerotherapy using other agents and after other surgical procedures as well as secondary rectal prolapse, including cauda equina syndrome, neural tube defects and sacrococcygeal teratoma, Hirschsprung disease, and congenital megacolon, were excluded from the study.

Data collection

After obtaining approval from the ethics committee of the hospital (IRB/2018/403/SIMS), all patients fulfilling the inclusion criteria were admitted to the pediatric surgery inpatient department of Services Hospital, Lahore. The whole procedure was explained and written consent was taken from the patients. Complete demographic information (name, age, sex, and address) was recorded in proforma. Patients were randomly divided into two groups with an equal number of candidates using the lottery method. Group A consisted of patients who were administered 5% phenol in almond oil and group B consisted of patients who were administered 15% hypertonic saline. All procedures were performed by a single surgical operating team to control bias.

**Figure 1:**
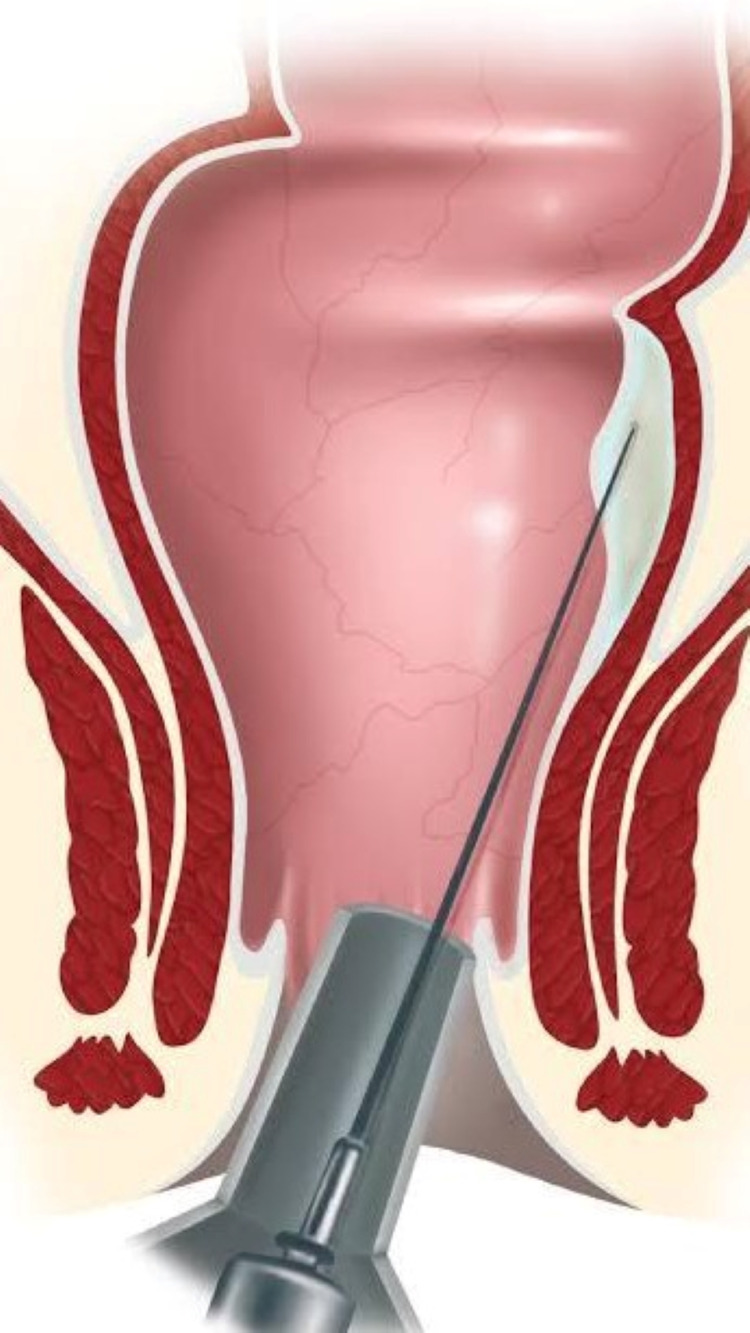
Injection with sclerosing material

Patients were admitted 24 h before the procedure to evacuate the bowel using saline enema. The patient was placed in the lithotomy position under general anesthesia. A 20-gauge spinal needle was introduced through the anal mucosa via a proctoscope or was externally introduced 2-3 cm from the anal margin, with a guiding finger in the anal canal, to a point several centimeters above the dentate line. Maximum amount of sclerosing agent used was 5-8 ml. It was circumferentially injected into the submucosal and perirectal space as the needle was withdrawn as shown in Figure 1. To prevent necrosis, bleeding, or stenosis, care was taken to avoid injecting the sclerosing agent into the mucosa. Patients receiving sclerosant injections were discharged the same day with simple analgesics and stool softeners. Patients were followed up for three months after surgery to note whether recurrence, incontinence, or anal stenosis occurred. The data were entered into the proforma.

Statistical analysis

Data were collated and analyzed using SPSS version 20.0 (IBM Corp., Armonk, NY, USA). Mean and standard deviation were calculated for quantitative variables such as age. The Chi-squared test was adopted by considering a p value of ≤0.05 as significant. Frequency and percentage were calculated for qualitative variables such as gender, recurrence rate, incontinence, or anal stenosis. Confounding factors, such as age and gender, were controlled through stratification. Post-stratification Chi-squared test was adopted by considering a p value of ≤0.05 as significant.

## Results

**Figure 2:**
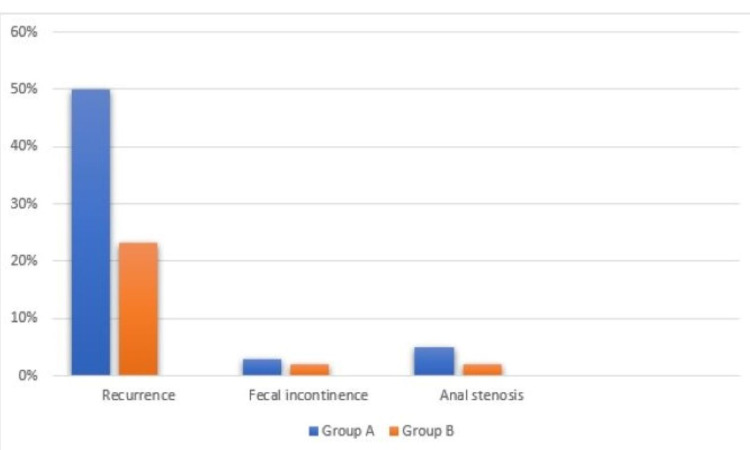
Comparison of efficacy and complication between the 5% phenol and 15% hypertonic saline groups

Our study included 120 children with idiopathic rectal prolapse. The mean ages of the patients were 3.97 ± 2.68 years in group A and 2.87 ± 1.84 years in group B, and most patients were between one and four years old, with 73.3% and 85% in groups A and B, respectively. This difference was not statistically significant (p=0.116).Gender distribution showed male dominance (71.7% in group A and 73.3% in group B).This difference was not statistically significant (p=0.838). A comparison of baseline characteristics (age and gender) between the two groups is shown in Table 1. Recurrence was observed in 50% of patients in group A and in 23.3% of patients in group B (p=0.002). Comparison of recurrence in both groups is shown in Table 2 and Figure 2. Stratification of recurrence according to age and gender in both groups is shown in Table 3.

**Table 1:** Comparison of baseline characteristics (age and gender) between the two groups

Age	Group-A (n=60)		Group-B (n=60)		P - Value
	No. of patients	%	No. of patients	%	
1-4 Years	44	73.3%	51	85.0%	0.116
4.1-12 Years	16	26.7%	9	15.0%	
Gender					
Male	43	71.7%	44	73.3%	0.838
Female	17	28.3%	16	26.7%	
Total	60	100	60	100	

**Table 2:** Comparison of recurrence in both groups

Recurrence	Group-A (n=60)		Group-B (n=60)	
	No. of patients	%	No. of patients	%
Yes	30	50%	14	23.3%
No	30	50%	46	76.7%
Total	60	100	60	100
p-Value	0.002			

**Table 3:** Stratification of recurrence according to age and gender in both groups

	Groups	Recurrence		Total	p-Value	
		Yes	No			
Age						
1–4 years	Group A	22	22	44	0.010	
	Group B	12	39	51		
	Total	34	51	95		
4.1–12 years	Group A	8	8	16	0.229	
	Group B	2	7	9		
	Total	10	15	25		
Gender						
Male	Group A	22	21	43	0.006	
	Group B	10	34	44		
	Total	32	55	87		
Female	Group A	8	9	17	0.188	
	Group B	4	12	16		
	Total	12	21	33		

Approximately 2% (n=3) of patients in group A and 2% (n=2) of patients in group B had postoperative fecal incontinence. This difference was not statistically significant (p=0.6478). In addition, 8% (n=5) of patients in group A and 2% (n=2) of patients in group B had anal stenosis. This difference was not statistically significant (p=0.2426) as shown in Figure 2. No toxicity of either drug (5% phenol or 15% hypertonic saline) was observed.

## Discussion

Rectal prolapse refers to the extrusion of some or all the rectal mucosa through the external anal sphincter. It is a benign condition characterized by partial or complete extrusion of the rectal mucosa or the rectal wall. Although various secondary causes have been described to result in rectal prolapse, most cases of rectal prolapse in childhood are idiopathic [11]. We used 15% hypertonic saline and 5% phenol in almond oil for injection sclerotherapy.

Hypertonic saline initiates a chemical inflammatory reaction that results in fibrosis around the rectal wall and perirectal tissue that causes the mucosa to adhere to the rectal wall and the rectum to adhere to the perirectal tissue, thereby preventing recurrence of prolapse. We used 15% hypertonic saline because it is easy to inject, can be easily prepared in a hospital laboratory, and is cost effective. This treatment can be performed as a day-care procedure.

As such, 5% phenol is an oil-based solution; it initiates an inflammatory reaction that results in fibrosis, which provides support to the tissue, thereby preventing recurrence of the prolapsed rectum. Injection absorption is slow. If accidentally injected into the vessels, 5% phenol can cause phenol toxicity that includes tonic-colonic convulsions, tachycardia, and increased blood pressure [12].

In our study, the mean age of the patients was 3.97 ± 2.68 years in group A and 2.87 ± 1.84 years in group B. In both groups, most patients were between one and four years old, with 73.3% and 85% in groups A and B, respectively. Sarmast et al. reported similar ages among patients with rectal prolapse in their study in 2015 [13]. Antao et al. found that the mean age of the patients was 2.6 years (range: four months to 10.6 years) [14]. Chaloner et al. also reported similar ages among the patients with rectal prolapse in their study [15]. In a study by Sadighi et al. from 1982 to 2000, 60% of patients belonged to the age group of two to six years; these results were also comparable to those of the present study [16].

Recurrence was observed in 50% of patients in group A (group that was administered with 5% phenol in almond oil) and in 23.3% of patients in group B (group that was administered with 15% hypertonic saline); p value was significant (0.002). Dolejs et al. found that all patients treated with phenol in almond oil and dextrose experienced recurrence [11]. At Liaquat University of Medical & Health Sciences, Jamshoro, Hyderabad, 22 children were subjected to injection sclerotherapy using 5% phenol in almond oil, and the cure rate was 72.73% after the first injection and 86.36% after the second and third injections. Only three out of these 22 (13.64%) patients were ultimately recommended surgery [17].

Hoque et al. used 5% phenol in almond oil in the treatment of rectal prolapse in children and found that 93.5% of the patients were cured after one injection and 99% were cured after two injections [18]. Wyllie also used 5% phenol in almond oil as a sclerosing agent and reported a cure rate of 91% after one injection and 100% after two injections [19]. Another study from Pakistan also reported a recurrence rate of 24% after the use of 5% phenol in almond oil as a sclerosing agent [10]. Our results were different from the three above-mentioned studies for group A with respect to recurrence; this may be because we conducted a single session for sclerotherapy. Repeated sessions with the same agent may cause some difference in recurrence rate.

Only a few studies that have used 15% hypertonic saline are available at present for the management of rectal prolapse in children for comparison. The recurrence rate in group B in our study was lower than that reported by Chaudhry et al. [10]. Our results were comparable to those of Abes and Sarihan who also used 15% hypertonic saline for the management of rectal prolapse in children; they reported that only one patient from among 16 children required a second treatment after the first injection [9]. Injection therapy is usually the first-line treatment at a majority of centers, and the recurrence following sclerotherapy is treated using repeat injection or a Thiersch procedure. Rectopexy is reserved for children who have recurrent rectal prolapse despite injection treatment and the Thiersch procedure.

The reason for a lower success rate and higher recurrence rate in our study could be that we used only a single injection, whereas in previous studies, injection sclerotherapy was repeated a second and then a third time. Bowel training is an important part of management of idiopathic rectal prolapse. Squatting position at defecation is not recommended. Similarly, timely evacuation of the bowel (defecation habit) is also stressed. The lack of counseling regarding behavioral modifications and introduction of a high-fiber diet in our population may be a reason for the suggested results. Moreover, this was a single-center study with a small sample size that was performed in a limited time, and further longitudinal studies are warranted in the local population.

## Conclusions

Based on these results, it was concluded that after a single injection, 15% hypertonic saline is a more effective sclerosing agent than 5% phenol in almond oil in terms of recurrence and has a statistically comparable rate of complication, including fecal incontinence and anal stenosis. Before referring them for surgery, injection sclerotherapy should be offered to all patients whose conditions do not show improvement with conservative treatment. Thus, 15% hypertonic saline injection can be safely selected as the sclerosing agent of choice in infants and children with idiopathic rectal prolapse.
